# Inflamma-MicroRNAs in Alzheimer’s Disease: From Disease Pathogenesis to Therapeutic Potentials

**DOI:** 10.3389/fncel.2021.785433

**Published:** 2021-10-28

**Authors:** Yuanyuan Liang, Lin Wang

**Affiliations:** Department of Emergency Medicine, Shengjing Hospital of China Medical University, Shenyang, China

**Keywords:** inflammation mediators, microRNAs, pathophysiology, diagnosis, therapy

## Abstract

Alzheimer’s disease (AD) is the most common cause of senile dementia. Although AD research has made important breakthroughs, the pathogenesis of this disease remains unclear, and specific AD diagnostic biomarkers and therapeutic strategies are still lacking. Recent studies have demonstrated that neuroinflammation is involved in AD pathogenesis and is closely related to other health effects. MicroRNAs (miRNAs) are a class of endogenous short sequence non-coding RNAs that indirectly inhibit translation or directly degrade messenger RNA (mRNA) by specifically binding to its 3′ untranslated region (UTR). Several broadly expressed miRNAs including miR-21, miR-146a, and miR-155, have now been shown to regulate microglia/astrocytes activation. Other miRNAs, including miR-126 and miR-132, show a progressive link to the neuroinflammatory signaling. Therefore, further studies on these inflamma-miRNAs may shed light on the pathological mechanisms of AD. The differential expression of inflamma-miRNAs (such as miR-29a, miR-125b, and miR-126-5p) in the peripheral circulation may respond to AD progression, similar to inflammation, and therefore may become potential diagnostic biomarkers for AD. Moreover, inflamma-miRNAs could also be promising therapeutic targets for AD treatment. This review provides insights into the role of inflamma-miRNAs in AD, as well as an overview of general inflamma-miRNA biology, their implications in pathophysiology, and their potential roles as biomarkers and therapeutic targets.

## Introduction

Alzheimer’s disease (AD) is one of the most common neurodegenerative disorders of the central nervous system (CNS) (Mangialasche et al., 2010). Its pathological features include neuroinflammatory plaques and neurofibrillary tangles, which are caused by abnormal deposits of amyloid-beta (Aβ) and hyperphosphorylated tau (p-Tau) proteins, respectively (Sun B. L. et al., 2018). The clinical manifestations of AD include cognitive dysfunction, psychological abnormalities, and an inability to perform simple everyday tasks that require some degree of cognitive acuity (Jacobs et al., 2018). The mechanisms that drive AD pathogenesis have not yet been fully elucidated. However, several hypotheses have been proposed, including the cholinergic neuron hypothesis, the Aβ toxicity hypothesis, and the Tau hypothesis (Ballard et al., 2011), among which the Aβ toxicity hypothesis is the most generally accepted. Aβ aggregation, especially Aβ42, is currently recognized as the main mediator of AD pathogenesis due to its ability to assemble into insoluble toxic fibrils that aggregate to form a neurotoxic β-fold lamellar structure. These structures progressively develop into neuritic plaques, which is an important driver of AD pathogenesis (Kim J. et al., 2009; Benilova et al., 2012). Aβ is thus considered a neurotoxic protein that activates complement and microglia and accelerates cell death through inflammatory responses (Keren-Shaul et al., 2017). Additionally, gliosis has been identified around senile plaques and neurofibrillary tangles (Terada et al., 2001). Aβ can also stimulate microglia to release inflammatory factors with strong neurotoxicity and promote the occurrence of inflammatory responses (Lueg et al., 2015). The onset and development of AD may thus result from the activation of inflammatory responses in the brain (Hansen et al., 2018). The potent immune response that follows Aβ stimulation may result in an indiscriminate damage of healthy nerve tissue, thus resulting in nerve injury and neuronal death (Ceccom et al., 2014). Moreover, inflammatory cytokines and lymphocytes entering the brain through the blood–brain barrier (BBB) can also trigger an inflammatory response in AD patients, and these pathological mechanisms are likely related to the effects of intercellular adhesion molecule-1 (ICAM-1) (Minogue et al., 2014; Solberg et al., 2014).

MicroRNAs (miRNAs) are a class of small endogenous non-coding RNAs approximately 18–25 nt in length (Hombach and Kretz, 2016). By interacting with the 3′ untranslated region (UTR) of target messenger RNA (mRNA), miRNAs degrade mRNA or inhibit protein translation and exert a negative regulatory effect. In addition to inhibiting gene expression, miRNA can also enhance gene expression, and its binding site in the target mRNA is not always limited to the 3′ UTR (Baek et al., 2008; Selbach et al., 2008). The specific expression of miRNA in immune cells suggests its role in regulating the proliferation, differentiation, and function of immune cells (Essandoh et al., 2016). Innate immunity is the first line of attack against bacteria, viruses, and other pathogens, and miRNA plays an important role in regulating innate immunity (Kumar and Bot, 2017). Recent studies have shown that miR-155, miR-146, and miR-223 play an important role in the regulation of acute inflammatory responses induced by pathogens *via* Toll-like receptors (TLRs) (Vegh et al., 2013). To study the regulatory role of miRNA in innate immunity, Taganov et al. (2006) examined the expression of 200 miRNAs in the THP-1 human monocytic leukemia cell line in response to lipopolysaccharide (LPS) exposure, and found that miR-146, miR-132, and miR-155 were upregulated in LPS-treated cells as compared to untreated cell. Interleukin 1 receptor-associated kinase 1 (IRAK1) and tumor necrosis factor receptor-associated factor 6 (TRAF6) are important adaptive molecules downstream of the TLR signaling pathway, which can cause the activation of nuclear factor κB (NF-κB) and activated protein 1 (AP-1) transcription factors, leading to increased cytokine release (Ghosh and Dass, 2016; Strickson et al., 2017). MiR-146a has a binding site on IRAK1 and TRAF6, and exerts a negative regulatory effect on this pathway (Williams et al., 2008). Interferon (INF)-γ/β can induce the upregulation of miR-155 in macrophages through autocrine and paracrine pathways of tumor necrosis factor α (TNF-α) (O’connell et al., 2007). MiR-155 also promotes the expression of TNF-α, suggesting that it plays a positive role in regulating the release of inflammatory factors in the innate immune response (Pedersen et al., 2009). miRNAs are also involved in adaptive immune responses such as immune cell activation, clonal proliferation, and antigen presentation. The upregulation of miR-181a can enable T cells to recognize inhibitory antigen peptides as active antigen peptides and enhance T cell signal transduction (Li et al., 2007). MiR-150, which is specifically expressed in mature lymphocytes, has been linked to the process of B cell differentiation. High expression of miR-150 in the spleen and thymus inhibits the differentiation of primary B cells into proprecursor B cells, thus affecting the formation of mature B cells (Zhou et al., 2007; Hu et al., 2018). However, miR-150 did not affect the formation of CD4 T cells, CD8 T cells, granulocytes, or macrophages (Lin et al., 2008). Several studies have confirmed that some miRNAs are involved in the regulation of inflammation, among which the most common ones include miR-21, miR-146a, and miR-155. Therefore, the term “inflamma-miRs” was coined to refer to these miRNAs (Quinn and O’neill, 2011). Further, additional studies have progressively linked miRNAs to the neuroinflammatory signaling, including NF-κB signaling (Amjad et al., 2019), TLR signaling pathway (Paschon et al., 2016), B cell receptor signaling (Borbet et al., 2021), and Jak/Stat signaling (Zhang M. et al., 2013).

Inflammation has been associated with all stages of AD pathogenesis, and the mechanisms that drive the inflammatory response intricately interact with other processes that jointly damage the nervous system and promote the onset and progression of AD. Therefore, the inflammatory response is not a passive system triggered by senile plaques and neuronal tangles during AD progression but rather an equally important pathogenic factor (Zhang B. et al., 2013). In this review, we summarized the most recent evidence for the involvement of inflamma-miRs in modulating the proinflammatory response in AD and further discussed the potential of circulating inflamma-miRs as biomarkers for the diagnosis and monitoring of AD progression, as well as the possibility of treating AD by regulating the expression of inflamma-miRs.

## Inflammation in Alzheimer’s Disease

Neuroinflammation plays an important role in the complex pathogenesis of AD. One of the main characteristics of this disease is the excessive activation of microglia, significant changes in neuronal morphology and function, and the production of a large number of inflammatory factors (Saito and Saido, 2018). Pathological studies of AD patients have demonstrated that a large number of microglia accumulate around and infiltrate the senile plaques, suggesting that microglia are closely related to AD progression (Mosher and Wyss-Coray, 2014). Aβ oligomers can activate pattern recognition receptors and related complements on the surface of microglia, triggering inflammatory responses (Chiarini et al., 2020). Activated microglia transform from a branched structure to an amoeba-like morphology and exhibit phagocytosis, which clears damaged or dead cells and Aβ (Tejera and Heneka, 2019). Additionally, continuously activated microglia can release a variety of inflammatory factors, which coincides with a significant decrease in the expression levels of Aβ-binding receptors and Aβ-degrading enzymes, as well as Aβ clearance capacity (Hickman et al., 2008). Reactive oxygen species (ROS) released by microglia serve as the second messenger to activate the NF-κB dependent signaling pathway, which can induce the production of a large number of inflammatory factors, thereby amplifying the inflammatory response and triggering a vicious cycle (Kempuraj et al., 2016). The pathological accumulation of tau protein is a hallmark of AD and related tau protein diseases. Maphis et al. (2017) demonstrated that the lack of the microglial fractalkine receptor CX3CR1 accelerated tau pathology and memory impairment. Additionally, recent studies in hTauCx3cr1(−/−) mice further confirmed that changes in the morphology of microglia may alter the brain microenvironment, which can drive tau pathology in a cell-autonomous manner and promote the propagation of misfolded tau proteins within anatomically connected regions of the brain (Maphis et al., 2015).

Microglia can express multiple types of recognition receptors to identify pathology-related and injury-related molecular patterns in the surrounding environment, thereby activating downstream signaling pathways in a cascade that leads to the maturation and release of neuroimmune inflammatory factors (Heneka et al., 2015). NLRP3 inflammasomes accelerate the progression of AD disease. Studies have shown that various neuroinflammatory factors are highly expressed in autopsy brain tissues of AD patients (Ozaki et al., 2015). More recent studies established a link between NLRP3 inflammasomes and AD pathology. Aβ can induce signal transduction associated with the NLRP3 inflammasome in microglia in an injury-specific molecular pattern to produce neuroinflammatory factors (Yang et al., 2019). Over-activated microglia produce a large number of neuroinflammatory factors, of which the extracellular superoxide nicotinamide adenine dinucleotide phosphate oxidase 2 (NOX2) was identified as the initiator of neuroinflammation-mediated neuronal degeneration (Tu et al., 2019). Ansari and Scheff (2011) found that NOX2 was upregulated in the frontal and temporal cortex of AD patients. Therefore, the upregulation of NOX2-related redox pathways is thought to be involved in the early pathogenesis and progression of AD (Ansari and Scheff, 2011). TLR2, TLR4, and Aβ are involved in the activation of microglia and neurodegeneration during AD (Balducci et al., 2017). Aβ activates microglia through TLR2 to produce neuroinflammatory factors, including TNF-α, IL-6, and interleukin-1β (IL-1β) (Kim C. et al., 2016). The Aβ-induced NF-κB signaling pathway also requires the involvement of TLR2 and TLR4, suggesting that TLRs play an important role in neuroinflammatory plaque deposition (O’halloran et al., 2014). The NF-κB signaling pathway plays a key role in the activation of microglia (Zusso et al., 2019). In patients with AD, activated NF-κB is mainly present in neurons and microglia surrounding neuroinflammatory plaques (Bronzuoli et al., 2016). Additionally, activation of the NF-κB pathway upregulates the expression of the β-site amyloid precursor protein cleaving enzyme 1 (BACE1) gene and promotes APP splicing to generate large amounts of Aβ (Jha et al., 2019). P38MAPK is involved in the signal cascade that controls cytokines and cellular stress response. Aβ activates p38MAPK and leads to an increase in the amount of calcium influx and ROS, which leads to increased mitochondrial oxidative stress and promotes AD onset and progression (Kheiri et al., 2018). Moreover, p38MAPK is also involved in the pathogenesis of AD by promoting Tau phosphorylation (Sun Y. et al., 2017), thus reducing synaptic plasticity (Beamer and Corrêa, 2021) and activating microglia to release pro-inflammatory factors (Liu Q. et al., 2019).

Astrocytes also play an important role in the pathogenesis of AD. In addition to being activated by Aβ, astrocytes are also activated by IL-1β released by microglia (Johnson et al., 2020). Activated astrocytes release a large number of inflammatory factors such as TNF-α, IL-1β, IFN-γ, and nitric oxide. The neurotoxic effects of these inflammatory factors damage neurons and are involved in a series of inflammatory responses, thus inducing Aβ accumulation (Zhao et al., 2011). In the brain tissues of AD patients, reactive astrocytes overexpress the mRNA of BACE1, which may contribute to Aβ plaque formation (Rossner et al., 2005). Additionally, activated astrocytes can overexpress the serine protease inhibitor α1-antichymotrypsin, which inhibits Aβ cleavage and acts as a neurotoxin that induces abnormal hyperphosphorylation of tau. Thus, α1-antichymotrypsin released by reactive astrocytes may play an important role in both the development of Aβ plaques and the generation of neuronal tangles (Padmanabhan et al., 2006).

In summary, neuroinflammation is an important mechanism of progressive neurodegeneration in AD. Therefore, regulating neuroinflammation may become a promising therapeutic strategy for AD treatment. Recent studies have shown that inflamma-miRs are dysregulated in neurons and have an important impact on cognitive function. These dysfunctional inflamma-miRs are related to the etiology and pathogenesis of AD (O’brien et al., 2013) and can directly or indirectly regulate Aβ and Tau expression. Due to the small molecular weight of miRNAs, they can pass through the BBB and are stably expressed in peripheral blood (Tominaga et al., 2015). Further, they can be isolated and tested with standard laboratory equipment, which greatly facilitates the analysis of miRNAs for clinical applications. Moreover, regulating the expression of inflamma-miRs may become a potential therapeutic strategy for AD treatment by affecting the inflammatory response during AD progression. Therefore, inflamma-miRs have broad prospects in the field of pathogenesis research, as well as in the diagnosis and treatment of AD. In the following sections, we will summarize and discuss recent studies on inflamma-miRs and their implications in AD detection and treatment.

## Role of Inflamma-MicroRNAs in Alzheimer’s Disease Pathogenesis

**FIGURE 1:**
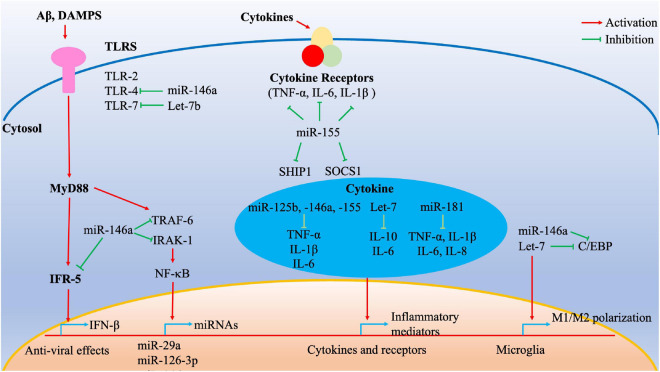
Cumulative effect of inflamma-miRNAs on inflammatory signaling pathways. Multiple inflamma-miRNAs may play a synergistic role in different inflammatory pathways. For instance, miR-146a targets different components of the MyD88/TLR/NF-κB pathways and microglia polarization. MiR-155 targets SOCS1 and SHIP1, whereas miR-125b, miR-146a, miR-155, Let-7, and miR-181 regulate multiple inflammatory mediators. C/EBP transcription factors are important for various inflammatory processes such as M1/M2 polarization and are targeted by Let-7. C/EBP, CCAAT/enhancer-binding protein; DAMPs, damage-associated molecular patterns; IFR, interferon receptor; IRAK, interleukin 1 receptor-associated kinase; NF-κB, nuclear factor κB; TLRs, Toll-like receptors; TNF, tumor necrosis factor; TRAF, tumor necrosis factor receptor-associated factor.

Given that the systemic pro-inflammatory state is associated with an increased risk of AD development and progression, our review focused on a subset of miRNAs that regulate the inflammatory processes (Figure 1 and Table 1). MiR-125b is widely expressed in a variety of human tissues and plays a key regulatory role in several biological processes (Ma et al., 2017). A previous study suggested that miR-125b overexpression could induce tau phosphorylation in primary hippocampal and cortical neurons of rat (Banzhaf-Strathmann et al., 2014). Further, miR-125b was significantly upregulated in cerebrospinal fluid (CSF) samples from patients with AD. Additionally, miR-125b also significantly increased the activities of TNF-α, IL-1β, and IL-6 in mouse neuroblastoma Neuro2a APPSwe/Δ9 cells. Conversely, IL-10 activity was markedly decreased in an AD *in vitro* model (Jin et al., 2018). Moreover, inhibition of miR-125b suppresses proinflammatory cytokines (TNF-α, IL-1β, and IL-6) (Zhuang et al., 2020). Therefore, miR-125b may also act as a pro-inflammatory factor to promote AD onset.

**TABLE 1:** The key inflamma-miRNAs involved in the pathogenesis of Alzheimer’s disease.

**miRNAs**	**Model/cell type**	**Expression**	**Pathological roles**	**References**
miR-125b	Hippocampal and cortical neurons of rat/neuroblastoma Neuro2a APPSwe/Δ9 cells	Up-regulated	Increases the activities of TNF-α, IL-1β, IL-6, acts as a pro-inflammatory factor to promote AD onset	Banzhaf-Strathmann et al., 2014; Jin et al., 2018
miR-29a	PBMCs in AD patients	Up-regulated	Inhibits the NF-κB signaling pathway	Sedighi et al., 2019
miR-126-3p	Cortical or hippocampal neurons from rat embryos	Up-regulated	Interferes with the neuroprotective effects of IGF-1 by downregulating the expression of PI3K and ERK pathway	Kim W. et al., 2016
miR-146a	Temporal cortex of AD patients/human THP-1 cells	Up-regulated	Involves in the negative feedback regulation of NF-κB activation; attenuates astrocytic inflammation; induces TLR tolerance in macrophages	Sethi and Lukiw, 2009; Nahid et al., 2011; Alexandrov et al., 2014
miR-155	3xTg AD mice	Up-regulated	Promotes microglia and astrocyte activation, increases production of inflammatory mediators such as IL-6 and IFN-β	Liu D. et al., 2019; Teter et al., 2019
Let-7	C57Bl/6J mice	Up-regulated	Acts both as extracellular signaling molecules and as ligands for TLR7 in microglia and neurons	Lehmann et al., 2012
miR-181	3xTg-AD mice	Up-regulated	Regulates the expression of Fos and SIRT-1	Rodriguez-Ortiz et al., 2014

*AD, Alzheimer’s disease; PBMC, peripheral blood mononuclear cell.*

The miR-29 family, which consists of miR-29a, miR-29b, and miR-29c, has been shown to be downregulated in AD (Hébert et al., 2008). There is an inverse relationship between miR-29a and the expression of the TNF-α receptor in AD (Zhao et al., 2017; Sedighi et al., 2019). Additionally, recent studies have shown that miR-29a mainly inhibits the NF-κB signaling pathway at the transcriptional level and targets key members of TNF-mediated pathways (Srivastava et al., 2016). Therefore, miR-29a may be involved in the occurrence and progression of AD by regulating the inflammatory response, however, the exact mechanisms of this process remain unclear.

MiR-126-3p modulates inflammation and innate immune responses by targeting NF-κB pathway components and endothelial adhesion molecules (e.g., VCAM-1) (Harris et al., 2008). MiR-126-3p has been reported to affect the expression of EGFL7, a secreted protein that regulates angiogenesis as a repair mechanism for neurodegenerative diseases and is also involved in adult neurogenesis. In the CNS, miR-126-3p is involved in regulating the insulin/IGF pathway and also regulates the vulnerability of neurons to toxic damage (Nikolic et al., 2010; Bicker et al., 2017). Other studies have demonstrated that elevated miR-126 levels increase Aβ42 toxicity in cell models and interfere with the neuroprotective effects of IGF-1 by downregulating the expression of PI3K and ERK pathway members (Kim W. et al., 2016).

MiR-146a is widely involved in the regulation of immune cells and its expression is localized in astrocytes and microglia. This miRNA is also involved in microglial polarization and is significantly upregulated in inflammatory activated microglia (M1 type) (Rom et al., 2010; Cunha et al., 2016; Liang et al., 2021). Further, miR-146a is also upregulated in the temporal cortex of AD patients and is involved in the negative feedback regulation of NF-κB activation (Alexandrov et al., 2014). Nakano et al. (2020) suggested that miR-146a upregulation in the hippocampus could attenuate astrocytic inflammation and may be a promising therapeutic agent for treating cognitive impairment in AD. Additionally, the targets of miR-146a include IRAK1, complement factor HCFH, and TRAF6, which are associated with the innate immunity of AD (Sethi and Lukiw, 2009). Moreover, miR-146a plays a major role in inducing TLR tolerance in macrophages. Upregulation of miR-146a induces TLR tolerance and alters the expression of inflammatory AD risk genes in response to LPS treatment in BV2 microglia (Nahid et al., 2011). Yang et al. (2021) demonstrated that an increase in miR-146a induced Aβ/LPS tolerance in microglia, leading to a decrease in Aβ clearance. Further, upregulation of miR-146a could inhibit the expression of the TLR4 signaling pathway and its related inflammatory genes NF-κB, IRAK1, and TRAF6, and reduce the release of inflammatory factors IL-1β, IL-6, and TNF-α, thus alleviating AD-associated neuroinflammation (Mai et al., 2019).

MiR-155 is considered a pro-inflammatory miRNA and has been shown to play a major role in the regulation of the innate immune response by regulating the production of cytokines and chemokines (Thai et al., 2007; Guedes et al., 2013). Previous studies have suggested that miR-155 is one of the key molecules in the inflammatory response of macrophages after TLR activation, and its upregulation depends on the JNK pathway (O’connell et al., 2007). The expression of miR-155 increased in LPS-stimulated microglia, which regulated the level of SOCS-1 and the production of cytokines and NO, indicating that miR-155 plays a pro-inflammatory role in both the peripheral immune system and the brain (Cardoso et al., 2012). Furthermore, miR-155 also participates in the gene regulatory network of astrocytes. The expression of miR-155 increases when astrocytes become activated and this miRNA is involved in the upregulation of pro-inflammatory cytokines in astrocytes by targeting SOCS-1 mRNA (Mor et al., 2011; Tarassishin et al., 2013). In the brains of 3xTg AD mice, miR-155 levels were strongly upregulated and coincided with an increase in microglia and astrocyte activation. Guedes et al. (2013, 2014) suggested that miR-155 and c-Jun were upregulated early in 3xTg AD mice and Aβ-activated microglia and astrocytes, thereby promoting the production of inflammatory mediators such as IL-6 and IFN-β (Teter et al., 2019). This effect is related to the miR-155-dependent decrease of SOCS-1. Furthermore, given that c-Jun silencing reduces the levels of miR-155 in Aβ-activated microglia and astrocytes, targeted regulation of miR-155 expression may be a promising strategy to regulate AD neuroinflammation (Guedes et al., 2014; Aloi et al., 2021). In addition to regulating glial cell function, miR-155 may be directly involved in the expression of inflammatory factors. Inhibition of miR-155 expression can attenuate the upregulation of TNF-α, IL-1β, IL-6, and their receptors, and substantially restore the impaired learning ability of AD rats (Liu D. et al., 2019).

Let-7 is an evolutionarily conserved miRNA family and nine Let-7 miRNAs are known to act as tumor suppressors and developmental regulators in humans (Lee et al., 2016). Let-7 miRNAs are also important regulators of the neuroinflammatory process and can also promote the anti-inflammatory M2 phenotype of microglia *via* targeted regulation of C/EBP-transcription factors (Cho et al., 2015). Let-7 miRNAs also promote astrocyte differentiation by targeting its negative regulators in glial progenitor cells and can also activate microglia by acting as damage-associated molecular patterns (DAMPs) against TLR7 (Lehmann et al., 2012). Additionally, Let-7 miRNAs regulate inflammation by targeting the cytokines IL-6 and IL-10 (Schulte et al., 2011; Teng et al., 2013). The proteins of the Let-7 family are released from neurons and are overexpressed in patients with AD (Slota and Booth, 2019). Another study reported that Let-7b miRNAs act both as extracellular signaling molecules and as ligands for TLR7 in microglia and neurons. Further, intrathecal Let-7b mediates neurodegeneration of the CNS (Lehmann et al., 2012).

The miR-181 family is highly expressed during the maturation of astrocytes and participates in the development of astrocytes. Overexpression of miR-181c in cultured astrocytes resulted in increased cell death upon LPS exposure. In TNFR1/TNFR2 double knockout mice, low miR-181 expression can enhance the production of pro-inflammatory cytokines (TNF-α, IL-1β, IL-6, and IL-8) induced by LPS, whereas miR-181 overexpression can significantly increase the expression of the anti-inflammatory cytokine IL-10 (Hutchison et al., 2013). Another study reported that miR-181 was upregulated in 3xTg-AD mice and directly regulated the expression of Fos and SIRT-1 (Rodriguez-Ortiz et al., 2014). However, its role in the inflammatory pathogenesis of AD has not been further investigated.

## Inflamma-MicroRNAs as Diagnostic Biomarkers for Alzheimer’s Disease

The diagnostic applicability of inflamma-miRNAs in AD has been investigated in previous studies (Table 2). For instance, AD patients were accurately distinguished from healthy controls based on the downregulation of miR-9-5p in whole-blood samples (Souza et al., 2020). Giuliani et al. (2021) evaluated the plasma levels of miR-17-5p, miR-21-5p, and miR-126-3p in a cohort of AD patients and found that they were significantly upregulated compared to those of healthy controls. Additionally, cases of mild and severe cognitive impairment could also be discriminated based on the level of miR-126-3p expression (Giuliani et al., 2021). Moreover, analyses of the expression profile of inflamma-miRNAs demonstrated that plasma miR-34a and miR-146a levels, as well as CSF miR-34a, miR-125b, and miR-146a levels in AD patients were significantly lower than those in control subjects. In contrast, the levels of CSF miR-29a and miR-29b were significantly higher in AD patients than those in control subjects (Kiko et al., 2014). The serum of AD patients also contained lower levels of miR-125b, with a specificity of 68.3% and a sensitivity of 80.8%. Interestingly, the levels of miR-125b were positively correlated to the outcomes of the Mini Mental State Examination (MMSE) in AD patients (Tan et al., 2014). Next-generation sequencing has also been used to quantify serum inflamma-miRNA levels for AD diagnosis. Guo et al. (2017) demonstrated that the serum miR-126-5p levels of AD patients were upregulated, whereas miR-181c-3p was downregulated, and both were positively correlated with the MMSE score. The expression profile of inflamma-miRNA in CSF could thus be used as an indicator for AD diagnosis. The levels of miR-29a were increased in the CSF of AD patients, with a sensitivity of 89% and a specificity of 70% [area under the curve (AUC) = 0.87] (Müller et al., 2016). Only one study has evaluated the prognostic role of inflamma-miRNAs in AD patients. Ansari et al. (2019) assessed the baseline blood levels of miR-146a and miR-181a in a cohort of patients with mild amnestic cognitive impairment and conducted new measurements after a 2-year follow-up. The authors demonstrated that miR-146a and -181a were upregulated in AD patients both at the baseline and after the 2-year follow-up. Moreover, higher levels of miR-146a were associated with the presence of the apolipoprotein E ε4 allele, coupled with a decrease in hippocampus volumes and CA1 neurons (Ansari et al., 2019).

**TABLE 2:** Diagnostic role of miRNAs in AD patients.

**MicroRNA**	**Expression pattern**	**Samples**	**ROC curve analysis**			**Clinical significance**	**References**
			**Sensitivity**	**Specificity**	**AUC**		
miR-17-5p miR-21-5p miR-126-3p	Upregulated	Plasma samples from 116 AD patients and 41 control individuals	– 55.0% 38.3%	– 70.7% 87.5%	– 0.68 0.62	AD diagnosis and assessment for development and progression of cognitive impairment in AD	Giuliani et al., 2021
miR-9-5p	Downregulated	Whole-blood samples from 36 AD patients and 38 control individuals	–	–	–	Diagnosis for late-onset AD	Souza et al., 2020
miR-29a	Upregulated	CSF samples from 18 AD patients and 20 control individuals	89.0%	70.0%	0.87	AD diagnosis	Müller et al., 2016
miR-34a miR-146a	Downregulated	Plasma samples from 10 AD patients and 10 control individuals	– –	– –	– –	AD diagnosis	Kiko et al., 2014
miR-34a miR-125b miR-146a	Downregulated	CSF samples from 10 AD patients and 10 control individuals	– – –	– – –	– – –		
miR-29a miR-29b	Upregulated		– –	– –	– –		
miR-125b	Downregulated	Serum samples from 105 AD patients and 115 control individuals	80.8%	68.3%	0.85	AD diagnosis and assessment the degree of cognitive impairment	Tan et al., 2014
miR-126-5p	Upregulated	Serum samples from 105 AD patients and 115 control individuals	72.7%	60.5%	0.72	Early diagnosis of AD	Guo et al., 2017
miR-181c-3p	Downregulated		71.9%	73.3%	0.78		
miR-146a miR-181a	Upregulated	Blood samples from 45 mild cognitive impairment patients	– –	– –	– –	Predicting the development of AD	Ansari et al., 2019

*AD, Alzheimer’s disease; AUC, area under curve; ROC, receiver operating characteristic.*

The development of cyclic inflamma-miRNAs as diagnostic biomarkers has an important theoretical and practical significance; however, several limitations must still be addressed. (1) miRNAs occur in very low concentrations in body fluids and the methods required for their separation and extraction are complicated. Additionally, mRNA is extremely prone to degradation, making it difficult to ensure the quality of the obtained miRNA. (2) There is a lack of unified and accurate detection methods for circulating miRNA. Each of the current methods for miRNA detection has its own limitations. For example, it is difficult to construct cDNA libraries for miRNA molecules with low abundance and tissue/temporal specificity. Further, qRT-PCR results are largely dependent on the design of primers and probes. The accuracy and repeatability of gene chip technology are poor, and this approach requires a relatively large initial sample size. In addition, high-throughput sequencing technology is expensive and time-consuming. (3) Studies on circulating miRNAs as diagnostic biomarkers are still in an exploratory stage, and multi-center and case-control studies are scarce. Moreover, the sensitivity and specificity of selected circulating miRNAs as possible diagnostic biomarkers require further verification. (4) Most importantly, the formation and action mechanisms of circulating miRNA are still unclear, and the reference value range of circulating miRNA under different physiological and pathological conditions has not been determined. However, as a diagnostic biomarker, circulating miRNA may soon substitute or supplement the current molecular indicators used to evaluate the occurrence and development of AD. Still, a substantial effort must be made to integrate our current knowledge of genomics, transcriptomics, proteomics, metabolomics, and systems biology to comprehensively clarify the molecular mechanisms that lead to AD emergence and development, as this would facilitate the development of more effective diagnosis, prognosis, and treatment methods.

## Therapeutic Potential of Inflamma-MicroRNAs in Alzheimer’s Disease

MicroRNAs play a key role in the pathogenesis of AD by regulating the expression of various genes and pathways, especially through neuroinflammatory mechanisms (Brites and Fernandes, 2015). The role of these inflamma-miRNAs in the pathogenesis and molecular processes of AD is generally quite complex. Given the central role of inflamma-miRNAs in regulating the molecular cascade in disease-associated and AD processes, they may become important therapeutic targets (Pogue and Lukiw, 2018). In fact, miRNA-based therapies have been recommended for a variety of neurological diseases such as cerebrovascular disease, amyotrophic lateral sclerosis, and Parkinson’s disease (Sun P. et al., 2018).

Regulation of miRNA expression *in vivo* is the basis of several therapeutic strategies, and various methods have been evaluated to explore and regulate their expression. For example, miRNA mimics are small synthetic double-stranded miRNA molecules that are processed into functional miRNAs, allowing for the high expression of functional intracellular miRNAs and inhibiting target mRNA expression (Rupaimoole and Slack, 2017; Sierksma et al., 2018). miRNA activity can also be inhibited, usually by delivering synthetic sequences that are complementary to the miRNA to block its binding to endogenous mRNA targets, such as antagonists (Jaber et al., 2019), targeted nucleic acid anti-miRNAs (Lukiw and Alexandrov, 2012), and miRNA sponges (Lu et al., 2019).

One of the major challenges of applying miRNA-based therapeutics to AD is the delivery across the BBB (Ha et al., 2016). Some miRNA delivery strategies with practical application prospects are being actively explored, and some progress has been made. These promising avenues include viral vectors, such as adenovirus and adeno-associated virus (AAV) vectors that can be used to induce miRNA expression in the CNS (Xie et al., 2015; Hordeaux et al., 2020). Non-viral delivery methods have also garnered increasing attention recently, including lipid- or polymer nanoparticle-based delivery systems that promote miRNA cellular uptake for therapeutic purposes (Yin et al., 2014; Bai et al., 2019). Therefore, the identification of optimal inflamma-miRNA therapeutic targets and the development of effective central neurotransmission systems for miRNAs will be key determinants of whether miRNA-based therapeutic strategies can enter clinical trials in the future.

## Conclusion

Pathological neuroinflammation is among the most important mechanisms of AD pathogenesis. Therefore, understanding the specific molecular processes that drive AD-associated neuroinflammation will undoubtedly facilitate the development of new diagnostic and therapeutic strategies to ameliorate the social burden of AD. Previous studies have reported that miRNAs can regulate neuroinflammatory signals. Some inflamma-miRNAs (e.g., miR-146a and miR-155) may be involved in several pathologic processes of AD and have been shown to play a central role in the control of inflammation. Additionally, inflamma-miRNAs reportedly exhibit significant differential expression in the peripheral circulation (plasma/serum and CSF) of AD patients. Therefore, miRNAs are promising biomarkers for AD diagnosis and prognosis, as well as potential targets for therapeutic purposes. Moreover, the induction or inhibition of inflamma-miRNAs may improve CNS tissue damage following AD-related neuroinflammation. Nevertheless, although some progress has been made in understanding the role of inflamma-miRNAs in neuroinflammation, multiple areas warrant future investigation. First, the mechanisms controlling miRNA levels and stability in neuroinflammatory signaling must be determined, including the processes by which mature miRNAs are degraded or cleared. Second, the ability of multiple miRNAs to target combinatorially a common pathway should be assessed. For instance, miR-126-3p, miR-146a, and miR-29 may synergistically modulate inflammation and innate immune responses by targeting NF-κB pathway. Third, miRNAs are being considered as a novel type of biomarkers and potential therapeutic targets for AD. The improvement in sensitivity and specificity could definitely promote the practical application of miRNAs as important biomarkers.

## Author Contributions

YL performed the literature searches and wrote the manuscript. LW critically revised the manuscript. Both authors made substantial and direct intellectual contributions to this work, and have approved it for publication.

## Conflict of Interest

The authors declare that the research was conducted in the absence of any commercial or financial relationships that could be construed as a potential conflict of interest.

## Publisher’s Note

All claims expressed in this article are solely those of the authors and do not necessarily represent those of their affiliated organizations, or those of the publisher, the editors and the reviewers. Any product that may be evaluated in this article, or claim that may be made by its manufacturer, is not guaranteed or endorsed by the publisher.
